# A Rare Case of Biliary Cast Syndrome After Orthotopic Liver Transplantation

**DOI:** 10.7759/cureus.85883

**Published:** 2025-06-12

**Authors:** Abdelrhman Refaey, Ahmed Attia, Ahmed Ebeid, Stephen Gray, Ameer Abutaleb

**Affiliations:** 1 Internal Medicine, Transplant Institute, George Washington University School of Medicine and Health Sciences, Washington DC, USA; 2 Internal Medicine, Holy Name Medical Center, Teaneck NJ, USA; 3 Internal Medicine, Howard University Hospital, Washington DC, USA; 4 Liver Transplant Surgery, Transplant Institute, George Washington University School of Medicine and Health Sciences, Washington DC, USA; 5 Gastroenterology and Transplant Hepatology, Transplant Institute, George Washington University School of Medicine and Health Sciences, Washington DC, USA

**Keywords:** biliary cast syndrome, biloma, graft rejection, ischemia, transplantation

## Abstract

Biliary cast syndrome (BCS) is a rare complication following orthotopic liver transplantation (OLT), characterized by the formation of casts within the biliary system. We present the case of a 48-year-old woman who developed BCS nine weeks post-transplant. The patient experienced multiple complications, including biloma formation, recurrent anemia, and septic shock. Endoscopic retrograde cholangiopancreatography (ERCP) revealed extensive biliary casts, which were partially removed using a SpyBite basket (Boston Scientific, Marlborough, MA, USA). This case highlights a rare but serious complication of liver transplantation. Early recognition and aggressive treatment are crucial for improving outcomes in liver transplant recipients with BCS.

## Introduction

Bile cast syndrome (BCS) is a rare complication of orthotopic liver transplantation (OLT) and was first described in 1975 [1]. It occurs in up to 4% to 18 % of OLT recipients [2]. They are solid formations composed of bile components, necrotic epithelial debris, fibrin, and sometimes microorganisms, which form within the biliary tree. They can obstruct bile ducts and lead to cholangitis, graft dysfunction, or failure, particularly after liver transplantation [3]. Its pathophysiology is still unclear but likely involves a blood supply-demand mismatch, such as a hepatic artery thrombosis or liver graft hilum narrowing [4]. We report a BCS case in a patient who underwent an OLT for alcohol use-induced acute on top of chronic decompensated liver cirrhosis.

## Case presentation

The patient is a 48-year-old female who underwent an OLT for alcohol-induced acute liver failure in the setting of chronic decompensated liver cirrhosis. Two weeks pre-transplant, she was admitted with worsening jaundice and fatigue with a model for end-stage liver disease (MELD) score exceeding 41. A CT scan of the chest, abdomen, and pelvis showed mild to moderate abdominopelvic ascites and worsening hepatosplenomegaly. 

Post-transplant, the patient received immunosuppression treatment, including methylprednisolone, mycophenolate mofetil, and tacrolimus. Two weeks later, she presented with lower abdominal pain with an elevated WBC count of 46,000. Her albumin level was 2.4 g/dL, total protein 4.7 g/dL, total bilirubin 3.5 mg/dL, alkaline phosphatase 420 U/L, and gamma-glutamyl transferase (GGT) 321 U/L. A CT abdomen/pelvis revealed a moderate volume of intraperitoneal fluid. A 10 Fr drain was placed into the left lower quadrant. A T-tube cholangiogram showed contrast leakage from the cystic duct remnant into the perihepatic space. 

**Figure 1:**
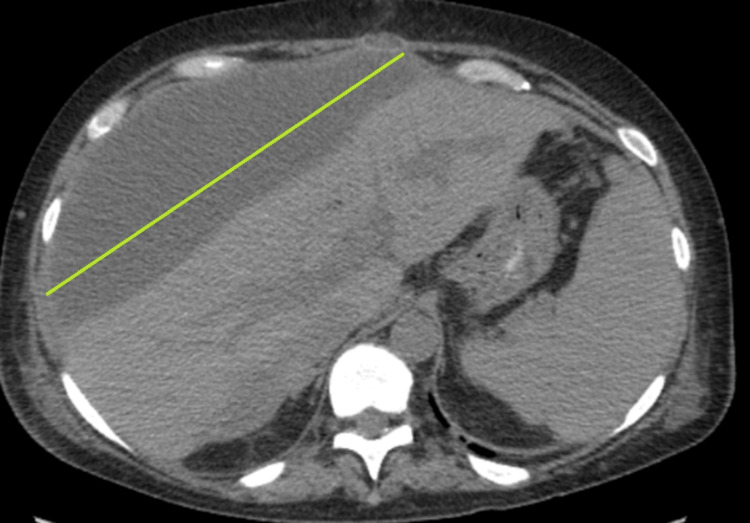
CT abdomen/pelvis showing a perihepatic collection measure of 25 cm (green line), indicating possible bile leak or biliary injury

Three weeks post-transplant, the patient was admitted for severe anemia (Hb 5.9 g/dl) for which she received three packed red blood cell (PRBC) transfusions. Per that admission, the CT abdomen/pelvis revealed a perihepatic collection measuring approximately 25 cm (Figure 1), consistent with biloma. A drain was placed into the bile collection, and a stent was deployed. Culture grew *Candida dubliniensis *for which fluconazole treatment was initiated. 

Four days later, she was readmitted for anemia with (hemoglobin (Hb) 6 g/dl) for which she received another three units of PRBC. Anemia workup revealed no acute blood loss, hemolysis, or nutritional deficiencies, and a low reticulocyte count raised concern for bone marrow suppression. An esophagogastroduodenoscopy (EGD) and colonoscopy were performed, showing a non-bleeding gastric ulcer. Ten days later, she experienced cardiac arrest with successful resuscitation. She was intubated and transferred to the ICU. 

**Figure 2:**
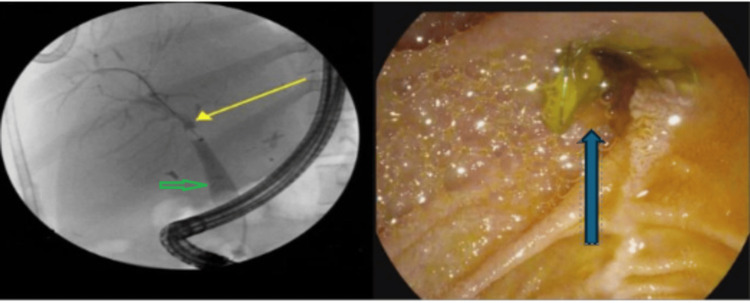
ERCP showing biliary anastomosis from a previous liver transplantation in the upper third of the main bile duct with mild stenosis (yellow arrow) along with dilated post-transplant donor duct to 12 mm with tapering proximally near the anastomosis (green arrow) and the right picture shows migration of the stent (blue arrow). ERCP: Endoscopic retrograde cholangiopancreatography Left frame: ERCP showing biliary anastomosis from a previous liver transplantation in the upper third of the main bile duct with mild stenosis (yellow arrow) along with dilated post-transplant donor duct to 12 mm, tapering proximally near the anastomosis (green arrow) Right frame: Migration of the stent (blue arrow)

The patient developed septic shock with positive blood cultures for methicillin-resistant *Staphylococcus epidermidis *(MRSE), vancomycin-resistant *Enterococcus* (VRE), and gram-negative bacteria. Treatment included linezolid, fluconazole, and valganciclovir. An endoscopic retrograde cholangiopancreatography (ERCP) was performed to address a possible bile leak and suspected ascending cholangitis. A proximally migrated biliary stent, which was partially occluded and lodged into the major papilla, was successfully removed intact (Figure 2). 

**Figure 3:**
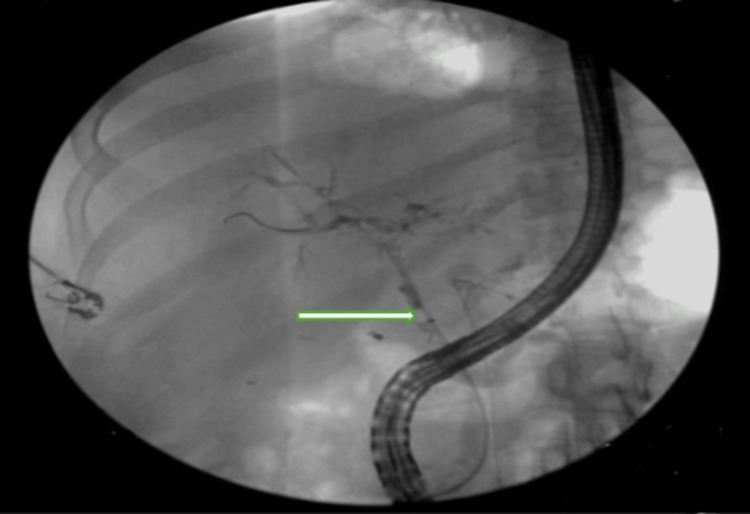
ERCP showing biliary system filling defect that extend to the left and right hepatic ducts and all intrahepatic branches (green arrow) ERCP: Endoscopic retrograde cholangiopancreatography

Two weeks later, the patient was admitted for worsening jaundice and abdominal pain. Laboratory values showed total bilirubin of 4.8 mg/dL, alkaline phosphatase of 948, GGT of 1254, aspartate transferase (AST) of 216 units/L, and alanine transaminase (ALT) of 350 Units/L. The ERCP revealed filling defects in the left and right hepatic ducts and all intrahepatic branches, indicative of biliary casts and stones (Figure 3). 

**Figure 4:**
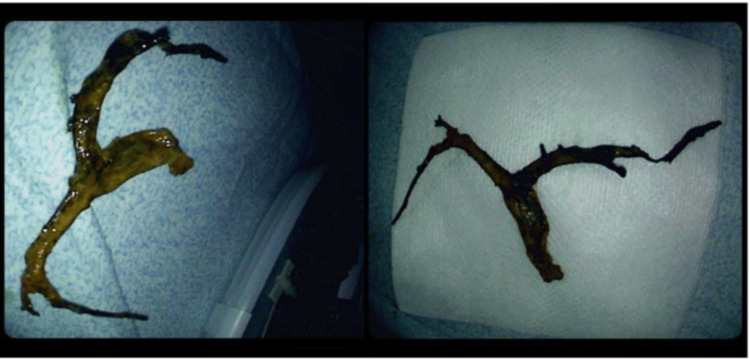
Gross picture of a large biliary cast after removal

**Figure 5:**
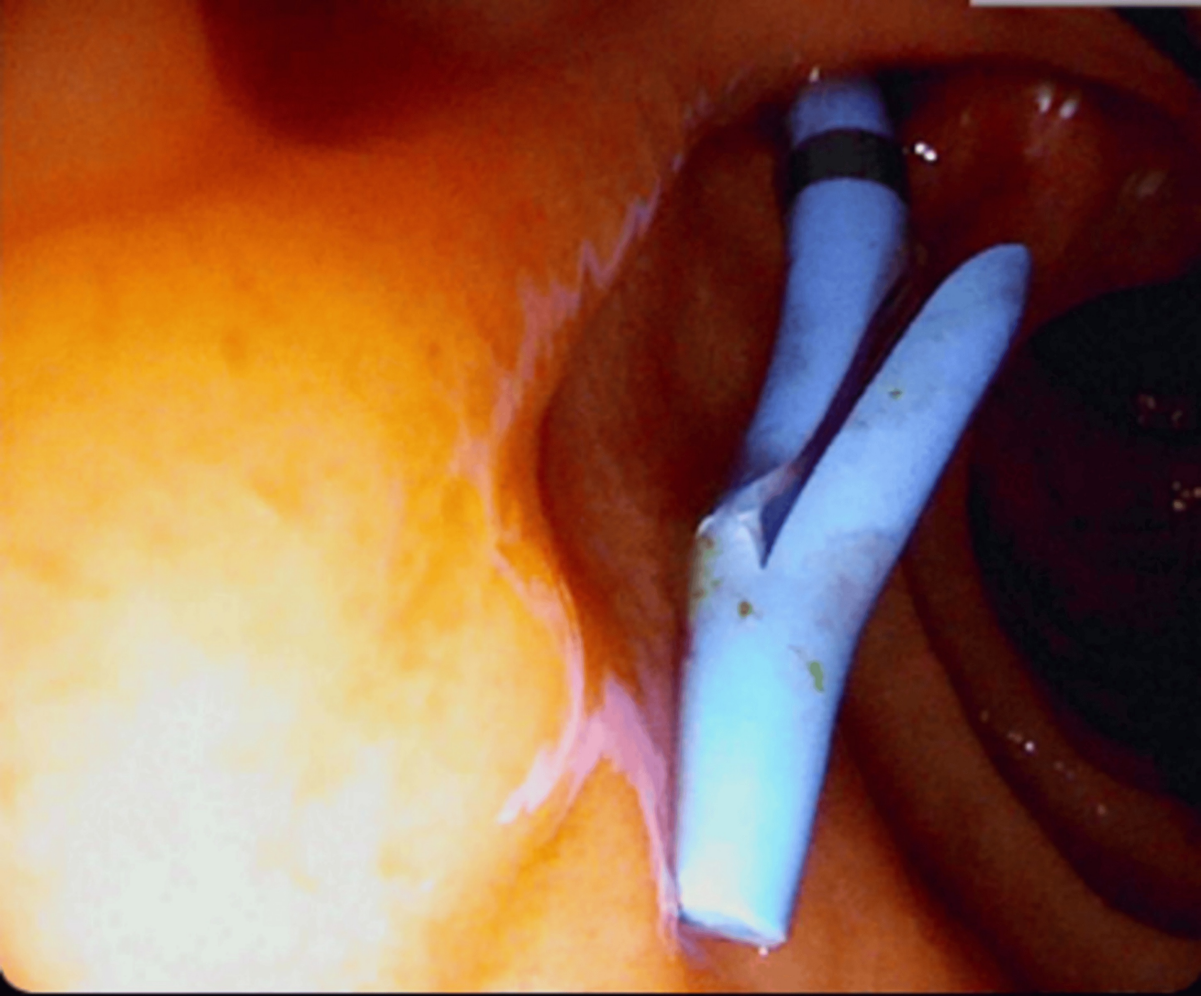
Intraoperative image of the 7 Fr by 12 cm trans papillary plastic biliary stent with a single external flap and a single internal flap placed into the right hepatic duct

**Figure 6:**
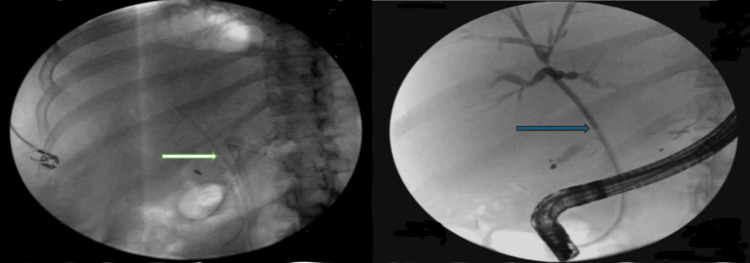
ERCP shows stent in a good position (green arrow) and normal flow of bile through the stent (blue arrow) ERCP: Endoscopic retrograde cholangiopancreatography

Electrohydraulic lithotripsy failed to remove the casts. A SpyBite basket (Boston Scientific, Marlborough, MA, USA) successfully swept a large cast from the right intrahepatic duct (Figure 4). A 7 Fr by 12 cm trans papillary plastic biliary stent was placed 11 cm into the right hepatic duct, with bile flowing appropriately through the stent (Figure 5). Post-procedural film showed an open lumen with the stent in a good position, and the bile flowing normally (Figure 6). 

## Discussion

Biliary cast syndrome is an uncommon post-liver transplant complication, characterized by casts and debris causing biliary obstruction with or without cholangitis. It occurs in 4% to 18% of liver transplant recipients and carries high morbidity and mortality rates up to graft rejection and repeated transplantation [1-6]. In two case series including a total of 1242 liver transplant recipients, reported rates of BCS were 3% [4,5]. It has been associated with limited graft survival, and some patients have undergone retransplantation [4]. 

On a molecular level, both biliary stone and biliary cast share almost similar biochemical structures (10% to 50% bilirubin and 5% to 10% protein, with less percentage of bile salt and cholesterol) and grossly, both appear as dark, solid material that encases the shape of the bile duct [4]. The pathophysiology involves chronic inflammatory cytokine stimulation leading to exudative inflammation of the bile duct epithelium. Consequently, fibrin deposits diffusely or locally within the bile duct, subsequently degenerating and binding with bile to form the structural framework of bile casts [7]. 

There is a multifactorial biliary duct pathology that may present with narrowing and or obstruction in the setting of ischemic liver injury, acute transplant rejection, or recurrent common bile duct (CBD) infection [8,9]. In our case, multiple risk factors may have led to the development of BCS, including recurrent severe anemia episodes that occurred three weeks post-transplant, where the hemoglobin level dropped to 5.9 g/dl. Additionally, the patient developed cardiac arrest and achieved return to normal circulation after 14 minutes of cardiopulmonary resuscitation (CPR). Both insults may have led to hepatic and biliary duct ischemia, increasing the risk of BCS development. 

The BCS usually presents within three months to one year from the transplant [4], which is consistent with the timeline of our case, which occurred nine weeks after the transplant date. Presentations range from asymptomatic to obstructive jaundice with or without acute cholangitis [6,10]. Our case presented worsening jaundice and abdominal pain, with labs showing an obstructive pattern. 

Several mechanisms could contribute to the development of biliary casts that mostly lead to hepatic and biliary strictures complicated by sludge or stone [11,12]. Interestingly, in our case, a CT scan revealed a perihepatic collection measuring approximately 25 cm, consistent with biloma. Based on that, a concern should be made about the possibility of the extra-biliary collection of bile being a predisposing factor to cast formation, as with the intra-biliary stricture. 

Based on current clinical practice, clearance of casts may be difficult to achieve with endoscopic intervention [13]. In a case series conducted by Shah et al., a combination of endoscopic and percutaneous methods was used to clear the casts in 60% of patients [4]. In a similar vein, we used electrohydraulic lithotripsy and a balloon together with the SpyBite basket to sweep the large cast.

## Conclusions

Biliary cast syndrome is a rare but serious complication of liver transplantation. Early recognition and aggressive management are crucial for improving outcomes. Further research is needed to better understand its pathophysiology and develop more effective treatment strategies.
